# Cognitive behavioural therapy for the treatment of late life depression: study protocol of a multicentre, randomized, observer-blinded, controlled trial (CBTlate)

**DOI:** 10.1186/s12888-019-2412-0

**Published:** 2019-12-27

**Authors:** Forugh S. Dafsari, Bettina Bewernick, Matthias Biewer, Hildegard Christ, Katharina Domschke, Lutz Froelich, Martin Hellmich, Melanie Luppa, Oliver Peters, Alfredo Ramirez, Steffi Riedel-Heller, Elisabeth Schramm, Magnus-Sebastian Vry, Michael Wagner, Martin Hautzinger, Frank Jessen

**Affiliations:** 1 Department of Psychiatry and Psychotherapy, University of Cologne, Faculty of Medicine and University Hospital Cologne, Kerpener Straße 62, 50931 Cologne, Germany; 2 Max-Planck-Institute for Metabolism Research, Gleueler Str 50, 50931 Cologne, Germany; 3 Department of Neurodegenerative Diseases and Geriatric Psychiatry, University of Bonn, 53105 Bonn, Germany; 4 Clinical Trials Centre Cologne, Gleueler Str 269, 50935 Cologne, Germany; 5 Institute of Medical Statistics and Computational Biology (IMSB), University of Cologne, Kerpener Str 62, 50931 Cologne, Germany; 6 Department of Psychiatry and Psychotherapy, Medical Center, University of Freiburg, Faculty of Medicine, University of Freiburg, Hauptstraße 5, 79104 Freiburg, Germany; 7 Department of Geriatric Psychiatry Central Institute of Mental Health, Medical Faculty Mannheim, University of Heidelberg, 68159 Mannheim, Germany; 8 Institute of Social Medicine, Occupational Health and Public Health, University of Leipzig, 01403 Leipzig, Germany; 9 Department of Psychiatry and Psychotherapy, Charité – Universitätsmedizin Berlin Campus Benjamin Franklin, Hindenburgdamm 30, 12203 Berlin, Germany; 10 Department of Clinical Psychology and Psychotherapy, Eberhard Karls University, Schleichstraße 4, 72076 Tuebingen, Germany; 11 German Center for Neurodegenerative Disease (DZNE), Sigmund-Freud-Str 27, 53127 Bonn, Germany

**Keywords:** Late-life depression, Major depression, Treatment, Psychotherapy, Randomized controlled trial

## Abstract

**Background:**

Late-life depression (LLD) is one of the most prevalent mental disorders in old age. It is associated with various adverse outcomes and frequent use of health care services thereby remaining a serious public health concern. Compared with depression in early adulthood, most treatment options of LLD are less effective. Psychotherapy may be particularly beneficial for LLD due to specific psychological conditions in old age and a low risk of side effects. Although cognitive behavioural therapy (CBT) is highly established and effective in depression in young and mid-life there is only a limited number of small studies on CBT in LLD. An LLD-specific CBT has not yet been compared to an active, but unspecific supportive psychological intervention in a multicentre trial.

**Methods:**

Here we present the design of the CBTlate trial, which is a multicentre, randomized, observer-blinded, active-controlled, parallel group trial. CBTlate aims at including 248 patients with LLD of both genders at 7 sites in Germany. The purpose of the study is to test the hypothesis that a 15-session individually-delivered CBT specific for LLD is of superior efficacy in reducing symptoms of depression in comparison with a supportive unspecific intervention (SUI) of the same quantity. The intervention includes 8 weeks of individual treatment sessions twice per week and a follow-up period of 6 months after randomization. The primary end point is the severity of depression at the end of treatment measured by the self-rated 30-item Geriatric Depression Scale (GDS). Secondary endpoints include depressive symptoms at week 5 and at follow-up (6 months after randomization). Additional secondary endpoints include the change of depressive symptoms assessed with a clinician-rating-scale and a patient reported outcome instrument for major depressive disorder, anxiety symptoms, sleep, cognition, quality of life, and overall health status from baseline to end-of treatment and to end of follow-up. Add-on protocols include MRI and the collection of blood samples.

**Discussion:**

This study is the first multicentre trial of a specific CBT intervention for LLD compared to an unspecific supportive psychological intervention administered in a specialist setting. It has important implications for developing and implementing efficient psychotherapeutic strategies for LLD and may be a significant step to broaden treatment options for people suffering from LLD.

**Trial registration:**

ClinicalTrials.gov (NCT03735576, registered on 24 October 2018); DRKS (DRKS00013769, registered on 28 June 2018).

## Background

Depressive disorders are frequent health problems and are among the leading causes of disability worldwide [1]. With the growing population older than 60 years of age and an increasing life expectancy, the demand for mental health care by the geriatric population is expected to increase. Depression is one of the most prevalent mental disorders in old age [2, 3]. Late-life depression (LLD) is generally defined as a depressive episode occuring after the age of 60 years [4]. A meta-analysis of epidemiological data suggests a prevalence rate of 7.2% (95% CI [4.4–10.6%]) for major depression and 17.1% (95% CI [9.7–26.1%]) for any depressive disorder or depressive symptoms in the elderly population [4]. The prevalence is about twice as high in women compared with men [4, 5]. According to a review of international studies, the incidence rates of LLD are 0.2–14.1/100 person-years [6].

LLD is associated with various adverse effects such as reduced quality of life, negative impact on physical comorbidities, functional impairment, and increased suicide as well as non-suicide mortality [7]. Suicide rates are highest in the high age group, particularly in males. While suicide occurs in 10.2/100.000 individuals in Germany below the age of 65, it increases to 25.7/100.000 after the age of 65 [8]. Moreover, LLD is a risk factor for all-cause dementia, including Alzheimer’s disease [9–13]. Depression increases the risk for other medical conditions such as cardiovascular disease and diabetes mellitus; it also accelerates the respective disease process and worsens outcomes [14]. It increases the socioeconomic burden of all of these medical conditions. Prospective analysis revealed that the health care costs of elderly individuals with depression are one third higher than those of non-depressed subjects [15, 16].

Overall, understanding and effectively treating depressive disorders in the elderly is of critical importance. Unfortunately, LLD is often misinterpreted as a physiological aging process and remains often under-recognized, misdiagnosed, and under-treated [17, 18]. Compared with depression in early adulthood, treatment options of LLD are limited. Antidepressants are less effective in LLD than in depression in young and in middle age adults [19]. The number-needed-to-treat (NNT) for remission of depression by antidepressant medication is around 7 in patients younger than 65 years of age [20] and increases to 14.4 in subjects older than 65 [21, 22]. Furthermore, side effects, intolerability and contraindications of antidepressants increase with age, which limits their application.

Psychotherapy is associated with less risk and potential benefit in LLD [22]. A highly established and effective type of psychotherapy for depression is the cognitive behavioural therapy (CBT), of which efficacy has been demonstrated in young and middle age adults [23]. To be effective in LLD it needs to be adapted to the specific needs and problems of older age patients, which are distinct from young and middle-aged adults (e.g. loss of significant others by death, loneliness, retirement, physical impairment, financial restraints, closeness of death).

The evidence of efficacy of LLD-adapted CBT is very limited. The vast majority of studies on CBT in LLD is of limited power or has other methodological shortcomings so that they either remain non-conclusive or are not generalizable. Meta-analyses on CBT in LLD include studies, which compared CBT against a waiting list control group [24–27]. Comparison against an active control intervention, however, is crucial to distinguish CBT-related effects from unspecific effects of intensified patient management and placebo response. Thus, waiting list control conditions are not sufficient to establish efficacy of a specific psychotherapeutic intervention. Studies with an active control group in LLD patients are single-centre studies with recruitment in the primary care setting and inclusion of self-referred patients, which may introduce bias and limited generalizability to clinical populations of the psychiatric care setting [24, 28].

In a single-centre pilot study, we tested the short- and long-term outcome of a manualized 15-session LLD-specific CBT-intervention in comparison to a manualized supportive, but nonspecific intervention (SUI), delivered either in an individual or a group setting [29]. LLD-specific CBT reduced depressive symptoms more than SUI. This effect was greatest in the CBT-arm with individual treatment and the effect extended over 1 year [29].

In the present study, we will test this intervention in the specialized clinical (psychiatric/psychotherapeutic) setting in patients with moderate to severe LLD in comparison to an unspecific supportive psychological intervention in a multicentre study with the aim to test the efficacy of CBT that is specific to LLD.

### Objectives

The primary objective of this study is to test the hypothesis that a 15-session individually-delivered CBT specific for late life depression is of superior efficacy in reducing self-reported symptoms of depression in comparison with a SUI of the same number of sessions and same duration. The secondary goals of the study are to test the efficacy of LLD-CBT in comparison with SUI on clinician-rated and patient reported outcome in major depressive disorder, anxiety, sleep, cognition, quality of life and overall health status. Furthermore, we will investigate the influence of childhood traumatic experiences and personality traits on the change of depressive symptoms. Additionally, blood sampling and MRI data will be acquired in order to investigate the underlying mechanisms in LLD and the specific effects of psychotherapy.

## Methods

### Study design

The investigator-initiated trial is registered as: “Cognitive behavioural therapy for the treatment of late life depression – a multicentre, randomized, observer-blinded, controlled trial (CBTlate)” at ClinicalTrials.gov (NCT03735576) and in the German Clinical Trials Register DRKS (DRKS00013769). It is funded by the German Federal Ministry of Education and Research (BMBF).

**Fig. 1:**
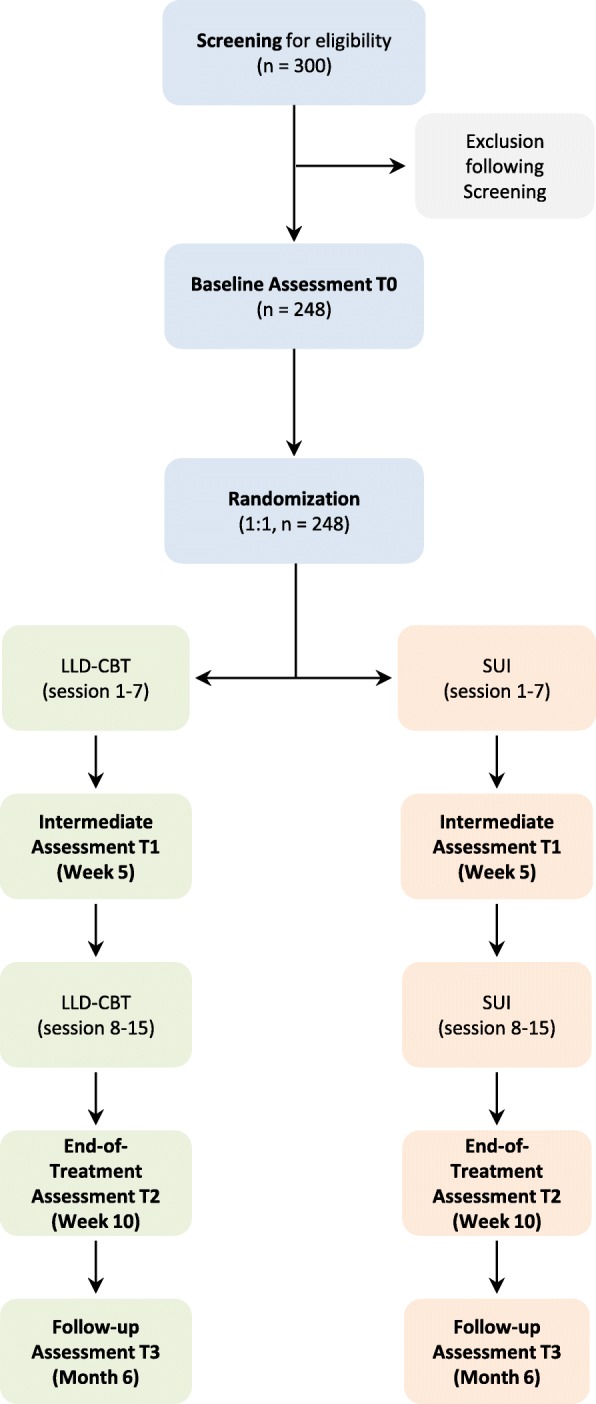
Trial Design. LLD-CBT: late-life depression cognitive behavioural therapy. SUI: supportive unspecific intervention

It is a randomized, multicentre, single blind (observer-blinded), active-controlled, parallel group trial in 248 patients with LLD of both genders at 7 trial sites in Germany. The intervention includes 8 weeks of manual-based, individual, 15-session, twice weekly, outpatient treatment for patients with LLD in each arm of the trial. Figure 1 illustrates the trial design.

The sequence of clinical and neuropsychological assessments is standardized across all sites. Furthermore, patients are asked to participate in additional procedures (blood sampling, MRI), which, however is not a requirement for participation. Four trial sites perform MRI and five sites participate in blood sampling. All data and biomaterial are stored centrally. All sites have a uniform procedure for intervention and control groups. Staff members at the participating sites conduct data and material acquisition according to SOP. The database and the sites are monitored centrally, including a query process.

This trial was approved by the Institutional Review Board/Institutional Ethical Committee (IRB/IEC) of the University of Cologne and by all other local IRB/IEC at the participating sites prior to initiation of the trial. Written informed consent is provided by all participants prior to any study procedure.

Adverse and Serious Adverse events (AE/SAE) are recorded in the case report form, reported to the project’s Data Safety and Monitoring Board (DSMB) and SAEs to each IRB/IEC. The DSMB will conduct regular phone conferences to identify potential safety concerns of the study and give advice on continuation or discontinuation of the trial.

### Data collection sites

The patients are recruited and treated at seven clinical sites in Germany, including the Department of Psychiatry and Psychotherapy at the University Medical Center Cologne, University Medical Center Bonn, Campus Benjamin Franklin Charité Berlin, University Medical Center Freiburg, Central Institute for Mental Health Mannheim, Department of Clinical Psychology and Psychotherapy at the Eberhard Karls University of Tuebingen and the Institute of Social Health, Occupational Health and Public Health (ISAP) at the University of Leipzig.

### Sample and setting

A total number of 248 participants with the diagnosis of moderate to severe depression (ICD-10) aged 60 years and older will be recruited at the seven participating trial sites. All participating sites have outpatient clinics and collaborate with networks of psychiatrist and psychotherapist, which will be involved in patient identification.

### Inclusion and exclusion criteria

Key inclusion criteria are:

- Out-patient status
- Male or female, age ≥ 60 years
- Ability to provide informed consent and written informed consent signed
- DSM-5 diagnosis of a Major Depressive Disorder/MDD (depressive episode at least moderate to severe according to ICD-10)
- Geriatric Depression Scale (GDS) score > 10
- Quick Inventory of Depressive Symptomatology – Clinician Rating (QIDS-C) score > 10
- Mini-Mental-Status-Test (MMST) score > 25
- No or stable (≥ 6 weeks) antidepressant treatment at baseline (medication will be kept stable at least throughout the 8 weeks of treatment)
- Sufficient German language skills

Key exclusion criteria include:

- Bipolar depression
- Schizophrenia or other psychotic disorders
- Substance abuse or dependency
- Dementia
- Acute suicidality (not only suicidal thoughts) assessed according to the Mini International Neuropsychiatric Interview (M.I.N.I. Version 7.0.2) Module B ‘Suicidality’ by the clinician and according to clinician practice guidelines; patients with suicidal ideations are eligible, as long as outpatient treatment is considered safe by the clinician
- Anxiety disorder as stand-alone diagnosis (e.g. generalized anxiety disorder, panic disorder, social phobia)
- Obsessive-compulsive disorder (OCD) as stand-alone diagnosis
- Participation in any other clinical trial parallel to this trial
- Additional psychological/psychotherapeutic treatment throughout 8-week treatment
- Regular use with scheduled daily dosing of benzodiazepines (not as needed) during 8-week treatment
- Severe or instable medical condition, which clearly impacts on depression or on the ability to participate in the trial
- Brain disease with severe functional impairment that impacts the ability to participate in the trial (e.g. aphasia, Parkinson’s disease)

### Interventions

The experimental intervention is a LLD-specific cognitive behavioural therapy (LLD-CBT) [30]. The control intervention (supportive unspecific intervention, SUI) is provided in the same quantity. The interventions are both provided over 15 bi-weekly sessions extending over 50 min each for a period of 8 weeks. They are delivered in individual face-to-face sessions at the respective trial sites. The LLD-CBT is guided by an adaptation of an already published treatment manual [30] and has been evaluated in a number of single-centre studies [29, 31, 32]. It is composed of 6 modules which correspond to the basic structure of CBT:

1. Therapeutic alliance building, identification of the patient’s history and problems, goal setting
2. Provision of the CBT rationale, development of a cognitive-behavioural model of the individual depression, life review, relating interventions to rationale and goals
3. Behavioural activation and day structuring
4. Cognitive techniques and thought control
5. Social and problem-solving skills
6. Relapse prevention, crises, and emergency plan

In contrast to CBT programs in young and middle age adults, all modules in the manual are tailored to address the characteristic needs of patients with LLD covering specific topics (experience of loss and physical impairment, life review and reminiscence elements). Therapists have to cover all modules but have flexibility how intensive each module for an individual subject is covered.

The alternative treatment (SUI) is an active and also manualized, but less specific control intervention, which has been successfully applied in the pilot study of this multicentre trial [29]. According to the not yet published SUI-manual, therapists will act as an empathic listener, who will not guide the session, but support the subject in him or her self-reflection and expression of emotions. SUI is a non-CBT approach and does not specifically address the characteristic needs of patients with LLD. SUI-manual defines acceptable and correct but also not acceptable therapist’s interactional behavior. All the therapists will be trained in both methods and deliver both interventions to avoid systematic effects of the therapist.

### Therapists training and monitoring of adherence

All study therapists will have a background of CBT-training and will be trained in a 2-day workshop on LLD-CBT and SUI. Before treating study patients, each therapist has to demonstrate his/her skills and adherence to the treatment protocol by treating two training subjects. All therapeutic sessions will be videotaped to assure adherence to the manuals in both treatment conditions. A random selection of treatment sessions per therapist will be evaluated by independent raters to monitor adherence to protocol. Adherence to treatment protocol will be evaluated by using a 20-item adherence scale (10 items LLD-CBT and 10 items SUI), which has been successfully applied in the pilot study of this multicentre trial [29]. Regular supervision will be offered twice a month by experienced and local study-licenced clinician and monthly by M.H. and his team in terms of conference calls plus personal meetings (at least twice a year). Supervision secures adherence to treatment protocols and keeping treatment quality on an overall high quality level.

### Assessments

#### Clinical and neuropsychological assessment

There will be a total of four assessments. The first visit of the study will be the screening and baseline (T0) visit (see Table 1). The screening and baseline assessment can either be performed in a combined visit or in two separated visits within the same week. The inclusion and exclusion criteria as well as the primary outcome will be assessed. The M.I.N.I. Version 7.0.2 will be conducted, which is a structured diagnostic interview developed for DSM-5 psychiatric disorders, assessing the 17 most common disorders in mental health [33–35]. A validated brief version of the M.I.N.I. is administered. Specifically, we assess the presence of a current, past and recurrent depressive episode (Module A), suicidality (Module B), manic/hypomanic episode and bipolar disorder (Module C), panic disorder, agoraphobia, social anxiety disorder, obsessive compulsive disorder, posttraumatic stress disorder and generalized anxiety disorder (Modules D – H and Module N), alcohol use disorder and substance use disorder (Modules I and J), psychotic disorder (Module K), medical, organic or drug caused disorders (Module O) and borderline personality disorder (Module Q). Only in those subjects who pass the screening procedure the secondary outcomes will be obtained. After the baseline assessment, the subjects will be randomized to either of the two treatment arms. After 7 therapy sessions, in the 5th week after randomization into trial, the primary and secondary outcomes will be assessed by the blinded rater. End-of-treatment primary and secondary outcomes will be obtained in week 10 (T2). The final follow-up assessment (T3) will be performed 6 months after randomization by the blinded rater.

**Table 1:** Outcome Assessments

	Screening (SCR)	Baseline (T0)	Intermediate (T1)	End-of-Treatment (T2)	Follow-up (T3)
	Week 1	Week 1	Week 5	Week 10	Month 6
Clinician assessment					
M.I.N.I. 7.0.2	•				
LIFE					•
QIDS-C	•	•^a^	•	•	•
Demographic data		•			
Neuropsychological assessment					
MMST	•				
CERADplus		•			•
NAB subtest maze		•			•
SCD Interview		•			•
Questionnaires					
GDS	•	•^a^	•	•	•
GAI		•	•	•	•
PRO-MDD		•	•	•	•
WHOQOL OLD/BREF		•	•	•	•
SF36		•	•	•	•
CTQ-SF		•			
ESS		•	•	•	•
ISI		•	•	•	•
RBDSQ		•	•	•	•
BFI-10		•			
Patient satisfaction				•	
Blood sampling		•	•	•	•
MRI		•		•	•

^a^GDS/QIDS-C at T0: only if T0 is performed later than 7 days after screening visit

All clinical interviews and all outcome assessments will be conducted by certified raters, who will be blinded to the treatment arm allocation. The raters are centrally trained and supervised in the application of all outcomes. They will have to participate in a 2-day training workshop including at least 2 videotaped completed assessments. Regular supervision by local study clinicians and central telephone conferences (at least monthly) will be performed to secure adherence to study protocol.

#### Blood sampling

Subjects will be asked to voluntarily participate in blood sampling. Blood samples are acquired at five sites. If the patient provides consent, the study physician takes venous blood (50 ml in total) from the subject at baseline (T0), T1, T2 and T3 for genetic and epigenetic analyses, measurement of Amyloid-β, Neurofilament light chain (NFL), Peripheral Blood Mononuclear Cells (PBMCs), Metabolomics, Proteomics and miRNA analyses. Blood samples include serum samples with clotting activator, EDTA plasma as well as EDTA whole blood and PAXgene RNA. All material is acquired, processed, stored and shipped to the central biorepository in Cologne according to SOP.

#### Magnetic resonance imaging

MRI data are acquired at four scanning sites. All sites operate Siemens scanners with MAGNETOM Prisma systems. An MRI of the subjects’ brain will be performed at baseline, end-of-treatment and follow-up to obtain a high-resolution structural T1-weighted image, a T2-weighted FLAIR image, a resting state fMRI, and diffusion tensor imaging (DTI) of the subjects’ brain. All data at each site will be conducted according to detailed SOP for the MRI acquisition.

### Outcome measurements

#### Primary outcome

##### Depressive symptoms

Symptoms of Depression will be measured with the *Geriatric Depression Scale (GDS)* [36]. The GDS is a widely established instrument to measure symptoms of depression in older subjects and to measure effects of treatment in clinical trials. It has also been employed as the primary endpoint in the pilot study [29]. It is a self-rating scale including 30 Items in a yes/no format. The GSD score can range from 0 to 30. The primary efficacy endpoint in this study is the change in the GDS score from baseline (T0) to end of treatment (T2).

### Secondary outcomes

#### Depressive symptoms

##### Quick-inventory of depressive symptomatology (QIDS-C)

The QIDS-Clinician-Rated Version (QIDS-C) includes 16 questions and is used to measure severity of the nine diagnostic symptoms of major depressive disorder (MDD) according to DSM-IV [37]. It was developed to provide equivalent weightings (0–3) for each symptomatic item and anchors that estimate the frequency and severity of symptoms of DSM-IV criterion items required to diagnose MDD [37–40]. The psychometric properties of the QIDS-C have been established in different studies.

##### Patient- reported outcome of major depressive disorder (PRO-MDD)

The German translation of a patient-reported outcome instrument (PRO) to assess symptoms of major depressive disorder is used [41]. As suggested by the Food and Drug Administration (FDA), patient-reported outcomes should be developed with input from patients and include data reported directly by the patient without interpretation of the patient’s response by a clinician [42]. The PRO instrument used in this trial is a validated depression-specific patient-reported outcome questionnaire that incorporates documented evidence of patient input in the development of the instrument. It assists in understanding and assessing MDD symptoms from patients‘perspectives at baseline as well as in evaluating treatment benefit during the psychotherapeutic intervention, at end-of-treatment and follow-up.

##### Longitudinal interval follow-up evaluation (LIFE)

A German translation and adaptation of the LIFE interview is performed at follow-up in order to assess the longitudinal course of depressive symptoms during the study period of 6 months. The LIFE is an integrated system for assessing the longitudinal course of psychiatric disorders. It is a semi-structured interview to collect detailed psychosocial, psychopathologic, and treatment information for a 6 month follow-up interval. The retrospective weekly psychopathological measures (“psychiatric status ratings”) are ordinal symptom-based scales with categories matching the symptoms of the DSM-IV diagnostic criteria. Psychosocial and treatment information are recorded and linked in time to the psychiatric ratings [43, 44].

#### Anxiety

##### Geriatric anxiety inventory (GAI)

In order to assess symptoms of anxiety at baseline and over the course of treatment the Geriatric Anxiety Inventory (GAI) will be used. The GAI is a validated 20-item scale that measures dimensional anxiety in older people. It is administered as a self-reported measure of symptom severity. It has also been shown to be sensitive to symptomatic change following intervention [45].

#### Quality of life

##### WHO-QOL-BREF and WHOQOL-OLD

To assess subjective quality of life the German version of the WHOQOL-BREF is used. It consists of the domains: physical and psychological aspects, social relationships, environment and overall quality of life. For assessing specific facets of quality of life in higher age, it is complemented by the 24-item add-on module WHOQOL-OLD. The WHOQOL-OLD is an instrument for the assessment of subjective quality of life in elderly people consisting of the six domains: sensory abilities, autonomy, past, present and future activities, social participation, (thoughts on) death and dying and intimacy [46]. Data showed good psychometric properties of the WHOQOL-OLD and suggest that it is an instrument well suited to identify the needs and wishes of an aging population [47].

##### Short-form health survey (SF-36)

The 36-item Short-Form Health Survey is a patient-reported survey measuring health-related quality of life. The SF-36 consists of eight subscales capturing vitality, physical functioning, body pain, general health perceptions, physical functioning, emotional functioning, social functioning and mental health. The SF-36 asks for the presence and severity of 36 Items over the course of the last week. It has been widely used and has good psychometric properties [48].

#### Cognition

##### Assessment of subjective cognitive functioning

A semi-structured interview regarding the details of subjective cognitive decline (SCD) will be administered and includes a series of questions regarding the presence, onset, course, and appraisal of problems with memory and other cognitive domains. This SCD interview was designed to capture the SCD-plus criteria [49], which are features of SCD that are supposed to be associated with increased likelihood of underlying AD pathology.

##### CERADplus

The Consortium to Establish a Registry for Alzheimer’s Disease (CERAD) neuropsychological test battery is applied in this study at baseline and follow-up to measure cognitive function [50–52]. It is a well-established used battery to assess individuals with neurocognitive disorders. In this trial the CERAD-Plus, which is complemented by three tests of executive functioning and mental speed (Trail Making Tests A and B, S-Words) is used [53]. German age, sex, and education-adjusted norms of the CERAD neuropsychological battery are available.

##### Neuropsychological assessment battery (NAB) maze subtest

The Neuropsychological Assessment Battery (NAB) is a comprehensive, integrated, modular battery of neuropsychological tests developed to assess a wide array of neuropsychological skills and functions in adults who have known or suspected neurocognitive dysfunction [54]. In this trial we specifically aim at complementing the CERADplus by assessing executive functions in older adults with the NAB maze subtest. We use the maze subtest of the NAB Screening Module in order to provide information on executive function at baseline and at follow-up.

#### Childhood trauma

The Childhood Trauma Questionnaire will be completed at baseline. The CTQ is a 28-item retrospective self-report questionnaire that measures the severity of the five categories of childhood trauma, which are emotional/physical/sexual abuse and emotional/physical neglect. It has been validated in terms of psychometric test properties in psychiatric patients [55]. The CTQ will be used to investigate the influence of childhood traumatic experiences on the course of the depressive symptoms and on treatment response.

#### Sleep

As sleep disturbances and/or the disruption of circadian rhythms are common symptoms of depression, they are measured in this study by self-report questionnaires. The Insomnia Severity Index (ISI) is a brief self-report instrument measuring the patient’s perception of both nocturnal and diurnal symptoms of insomnia. The ISI was developed as a patient reported outcome measure intended both for screening and for assessing the efficacy of treatment. It has been validated in numerous studies with patients with insomnia [56, 57]. The Epworth Sleepiness Scale (ESS) is a self-administered questionnaire with 8 questions measuring the patients’ level of daytime sleepiness, or their average sleep propensity in daily life [58–60]. The psychometric properties of the ESS have been investigated widely. The REM Sleep Behavior Disorder Screening Questionnaire (RBDSQ) is a specific questionnaire developed to assess the most prominent clinical features of REM sleep behavior disorder (RBD) according to the International Classification on Sleep Disorders. It is a 10-item, patient self-rating instrument with good psychometric properties [61].

#### Personality

The Big Five-Inventory 10 Item Short Version (BFI-10) is a 10-item scale measuring the personality traits Extraversion, Agreeableness, Conscientiousness, Emotional Stability, and Openness. The scale was developed based on the 44-item Big Five Inventory and designed for contexts in which respondents’ time is limited [62]. It is used at baseline to assess the influence of personality traits on treatment outcome.

#### Socio-demographic and medical data

In the initial screening visit demographic data, concomitant medication and diseases will be documented. Subjects, who are eligible to participate in the study, will undergo an assessment of sociodemographic data, medical history as well as psychiatric and psychotherapeutic history. Medical data concerning previous and present diseases, inpatient and/or outpatient psychiatric/and or psychotherapeutic treatments and suicidal attempts will be obtained at baseline and at follow-up.

#### Process evaluation

After completion of the 8-week psychotherapeutic intervention the patient satisfaction as well as the needs and goals met and not met by the intervention will be assessed by an adapted version of the patient satisfaction questionnaire ZUF-8. The ZUF-8 is a brief and reliable instrument to measure general satisfaction with treatment [63].

### Measures taken to minimize/avoid bias

#### Randomization

This trial is a controlled trial with randomization. Subjects are assigned to treatment arms (1:1 randomization) by means of the central 24–7 internet randomization service ALEA, hosted by the Institute of Medical Statistics and Computational Biology (IMSB) at the University of Cologne. Randomization to either of the two treatment arms will be performed as stratified block randomization. The allocation sequence is made from permuted blocks of varying length, in which block size is unknown to the investigators. The randomization will be stratified by trial site in order to prevent unbalanced allocation of the intervention of interest and the control intervention to sites. In the rare case of unavailability of the service, a fax-based fallback procedure is used. When all data have been collected, cleaned and the database locked, the biostatisticians will be given access to the randomization code.

#### Blinding

This study is a single-blind (observer-blinded) trial. All clinical ratings will be completed by a trained and independent rater, who will be blinded to the treatment allocation. Each of the sites implements procedures to obscure the treatment assignment from the raters, who complete the clinical ratings. This will be warranted by: 1) informing and reminding the patients at each visit not to mention anything that might reveal their treatment condition to the rater and 2) locating the rater and the study therapists at different physical locations.

#### Control of adherence bias

Performance bias is minimized by carefully following the study protocol and continuous monitoring of adherence. Both the experimental and the control intervention are manual-based and are provided by the same therapist, trained in both programs. Each therapist will conduct both treatments (CBT and SUI). All therapists are required to adhere to the therapy manuals which include the therapeutic procedures and interventions. Adherence to manuals in both treatment conditions is continuously supervised by videotaping of all therapy sessions and by external and central assessment of the videotapes on a randomized basis using adherence scales.

#### Control of confounding factors

Patients can not engage in any off-study psychotherapeutic treatment (e.g. outpatient psychotherapy) or other psychiatric intervention (e.g. TMS, tDCS, ECT, VNS) during the 8 week treatment period. In case of existing pharmacological treatment the antidepressive medication has to be stable for at least 6 weeks at baseline and stable during the 8 week treatment period. Due to ethical reasons, it is not possible to prevent any change in medication or other types of treatment until the month 6 follow-up visit.

### Statistical methods

#### Power calculation

The sample size is calculated for the primary hypothesis testing superiority of LLD-CBT against SUI with regard to the change of GDS scores from baseline to end-of-treatment assessment in week 10. The power calculation is based on the results of the pilot trial [29]. In the pilot trial the experimental group (*n* = 27) improved from 19.26 ± 3.92 to 10.67 ± 6.42 (mean ± SD) and the control group (*n* = 25) from 20.68 ± 3.96 to 14.92 ± 7.58 on the GDS. Thus, assuming correlation of 0.5 between measurements, a standardized difference of about 0.52 (≈2.83/5.47) was observed between groups of the pilot study [29]. The present multicentre trial was powered to detect a clinically relevant difference of 2.5 GDS points (d = 0.4). The two-sample t-test requires 99 subjects per group to reach 80% power at two-sided significance level 5% to detect such difference. Accounting for up to 20% attrition from baseline to follow-up, 124 (≈99/0.8) subjects per treatment arm are needed, i.e. 248 subjects in total. Adjusting for baseline in a mixed model for repeated measures (MRM) approach is likely to further increase the statistical power. Due to stratification by therapist, the treatment effect is not affected by between-therapist variation [64].

#### Statistical analyses

The primary (full) analysis set (FAS) is derived from the intention-to-treat principle (all subjects randomized with a valid baseline assessment and at least one valid follow-up outcome assessment). The primary outcome measure „change in GDS from baseline to end-of-treatment “is evaluated by a mixed model for repeated measures (MMRM) with fixed effects baseline, therapist, group, time and the interaction group*time (ARH1-structured covariance matrix over time) with corresponding marginal means and contrast tests. Data from low recruiting therapists (i.e. < 4 subjects) are pooled by study centre. The potential clustering of observations of participants by therapist or centre will be investigated by multilevel modelling [65]. Since mixed models can be expected to yield valid results only in case of missingness-at-random, multiple imputation approaches are taken to assess the robustness of the results. Specifically, missing values due to death, illness or chance are separately imputed assuming mixtures of missingness-not-at-random patterns. Imputation data sets are post-processed by multiplication with factors and addition of offsets (tipping point analysis) [66].

Secondary outcomes (i.e. further time points and measures) are analyzed along the same lines, i.e. using mixed modelling (or GEEs). Time-to-event (e.g. remission, drop-out or survival) distributions are summarized by the Kaplan-Meier method and compared by the (stratified) log-rank. Analysis of the set of subjects observed and treated per protocol (PP) defined as a subset of the FAS population (all subjects without major protocol violations and at least 9 sessions in one of the therapeutic intervention as scheduled in the trial protocol) is supportive; similarly the compliers’ average causal effect (CACE) is calculated [67]. Subgroup analyses are done by study site and gender. Quantitative outcomes are summarized by mean, standard deviation and percentiles (i.e. 0, 25, 50, 75, 100), qualitative outcomes by count (percentage). Demographic data will be described overall with respect to all relevant populations. For age, weight, height, and body mass index descriptive statistics (n, mean, standard deviation, minimum, Q1, median, Q3, maximum) will be provided. For the categorical variables (e.g. ethnic group, gender) absolute and relative frequencies will be tabulated. All safety and tolerability parameters (adverse events, serious adverse events) will be aggregated (using absolute and relative frequency) and listed by subject, treatment group, category, seriousness, severity, relatedness, respectively. All analyses will be performed for the safety population and stratified by treatment group. The safety population is defined as all subjects with at least one treatment.

## Discussion

This multicenter, randomized, observer-blinded, controlled trial will examine the efficacy of an LLD-adapted CBT in comparison to an unspecific supportive intervention (SUI) in 248 patients with LLD. It is the largest and first confirmatory multicenter trial to test the hypothesis that an LLD-specific cognitive behavioral therapy is superior to an unspecific supportive intervention in patients with late-life depression with regard to reducing symptoms of depression over the course of the treatment and at 6 months follow-up as one secondary endpoint. A major strength of the study is that it addresses the unmet medical problem of insufficient treatment of LLD. The target population will be patients with moderate to severe disease recruited from the psychiatric setting. This will ensure targeting the clinically most relevant patient group and is distinct from earlier studies, which included self-referred patients or only mildly depressed patients. If effective, this treatment could be provided and relieve the burden of depression in terms of reducing symptoms of depression, potentially associated morbidity and mortality, including the negative impact of depression on several other medical conditions, and improving quality of life for this patient group. These potential effects are of particular importance in the rapidly growing patient group above the age of 60 years. The present trial does not focus on the comparable efficacy of CBT vs. medication or placebo, which might be seen as a limitation. Given the long duration of the trial it would be unethical to keep nonresponsive depressed patients on medication alone or in a placebo condition. Due to ethical reasons, it is also not possible to prevent any change in medication or other types of psychological treatment after the 8-week treatment period until the month 6 follow-up visit.

In addition to the main analysis described above, this study will be able to address a number of other important research questions. We will investigate the influence of pre-specified baseline characteristics on the main outcome measure as well as on key secondary outcomes, particularly of baseline symptom severity, chronicity as well as depression subtypes related to age of depression onset (early-onset vs. late-onset depression).

Further, a substantial number of studies have linked depression to cognitive impairment [9, 11, 13]. The inter-relationship between these clinical entities is complex and not conclusively understood. Therefore, we will focus on cognitive aspects of LLD at baseline and the effect of neuropsychological deficits on treatment response over the course of the psychotherapeutic interventions.

Neuroimaging studies have revealed patterns of structural and functional abnormalities of frontolimbic regions as well as disruptions of frontolimbic white matter tracts in DTI studies of LLD patients [68–73]. Additional MRI analysis will deal with specific effects of psychotherapy on the resting state and structural connectome in the aging brain and the effect of specific white matter pathway lesions on treatment outcome.

Blood sampling will enable genetic and epigenetic analyses, the measurement of Amyloid-β and NFL, PBMCs, Metabolomics, Proteomics and miRNA analyses to allow for evaluation of biological predictors and mechanisms of treatment response.

The study will have an impact on clinical practice. There is a marked gap in psychotherapeutic treatment in patients over the age of 60 years. If this trial demonstrates a convincing evidence of LLD-CBT efficacy, the currently insufficient provision of psychotherapy to patients in the higher age range may be improved. Solid evidence of efficacy for LLD-CBT would facilitate implementation of this treatment in health care.

## Data Availability

Not applicable.

## Abbreviations

AE: Adverse event
CBT: Cognitive behavioral therapy
DMSB: Data Monitoring and Safety Board
EC: Ethics Committee
ECT: Electroconvulsive Therapy
GCP: Good Clinical Practice
LLD: Late-life depression
miRNA: Micro RNA
NFL: Neurofilament light chain
PBMC: Peripheral Blood Mononuclear Cell
SAE: Serious adverse event
SUI: Supportive unspecific intervention
tDCS: Transcranial Direct-Current Stimulation
TMS: Transcranial Magnetic Stimulation
VNS: Vagus Nerve Stimulation
